# MiRNA-Mediated Control of HLA-G Expression and Function

**DOI:** 10.1371/journal.pone.0033395

**Published:** 2012-03-16

**Authors:** Irit Manaster, Debra Goldman-Wohl, Caryn Greenfield, Daphna Nachmani, Pinchas Tsukerman, Yaron Hamani, Simcha Yagel, Ofer Mandelboim

**Affiliations:** 1 The Lautenberg Center for General and Tumor Immunology, IMRIC, Hebrew University Hadassah Medical School, Jerusalem, Israel; 2 Department of Obstetrics and Gynecology, Center for Human Placenta Research, Hadassah-Hebrew University Medical Centers, Mt. Scopus, Jerusalem, Israel; Institut Jacques Monod, France

## Abstract

HLA-G is a non-classical HLA class-Ib molecule expressed mainly by the extravillous cytotrophoblasts (EVT) of the placenta. The expression of HLA-G on these fetal cells protects the EVT cells from immune rejection and is therefore important for a healthy pregnancy. The mechanisms controlling HLA-G expression are largely unknown. Here we demonstrate that miR-148a and miR-152 down-regulate HLA-G expression by binding its 3′UTR and that this down-regulation of HLA-G affects LILRB1 recognition and consequently, abolishes the LILRB1-mediated inhibition of NK cell killing. We further demonstrate that the C/G polymorphism at position +3142 of HLA-G 3′UTR has no effect on the miRNA targeting of HLA-G. We show that in the placenta both miR-148a and miR-152 miRNAs are expressed at relatively low levels, compared to other healthy tissues, and that the mRNA levels of HLA-G are particularly high and we therefore suggest that this might enable the tissue specific expression of HLA-G.

## Introduction

HLA-G is a nonclassical HLA class Ib molecule that possesses unique features, such as low polymorphism, restricted expression and seven spliced isoforms [1], [2], [3]. HLA-G expression is restricted mainly to the EVT cells of the placenta, which is of fetal origin. The EVT cells invade the uterine mucosa (the decidua) and the spiral arteries [4], [5]. The restricted expression of HLA-G is thought to provide immunoprotection to the semiallogenic embryo, especially from the decidual NK cells, which are the major lymphocyte population at the fetal-maternal interface, early during pregnancy [4].

NK cell activity is balanced by signals from activating and inhibitory receptors and in that regard, HLA-G inhibits NK activity mainly via the interaction with the inhibitory receptor LILRB1 [6], [7]. LILRB1 has a higher affinity to HLA-G than other HLA class-I molecules due to increased avidity formed as a result of HLA-G disulfide-dimers and trimers [6], [7]. HLA-G also binds the inhibitory receptor KIR2DL4 with very low affinity, however, the necessity of KIR2DL4 for reproductive success has been questioned [8], [9], [10]. HLA-G is important for a successful pregnancy and low expression levels of HLA-G at the fetal-maternal interface are associated with pregnancy complications, such as preeclampsia [11]. It is also expressed by tumors, virus-infected cells and by transplanted organs [12]. The restricted expression of HLA-G and the fact that HLA-G on the one hand is important for successful embryo implantation and fetal survival but, on the other hand, being potentially detrimental in tumors and viral infections suggest that the expression of HLA-G is tightly regulated [13]. However the mechanisms controlling HLA-G expression are currently largely unknown.

Although the HLA-G protein shows low polymorphism in the human population, especially when compared with other HLA class-I molecules, it possesses high polymorphism in its promoter region and in its 3′untranslated region (UTR). A 14 bp insertion/deletion polymorphism in the 3′UTR of HLA-G has been associated with RNA stability, however it is not clear whether or not this polymorphism can indeed affect HLA-G expression [13]. It has been suggested that the expression of HLA-G is influenced by a C/G polymorphism at position +3142 of the gene, which resides in the 3′UTR of HLA-G [14]. This site was also suggested to be a site targeted by some miRNAs [15] and the presence of guanine instead of cytosine at this position was claimed to favor HLA-G 3′UTR targeting by miRNAs.

MiRNAs are ∼22 nt long non-coding RNA molecules that usually negatively regulate gene expression post-transcriptionally, mainly by binding to the 3′UTR of mRNAs [16], [17]. Such binding results in either translational inhibition or in mRNA degradation [16], [17]. It is now known that miRNAs play a pivotal role in many biological and pathological processes [18]. Three miRNAs were suggested to target the 3′UTR of HLA-G: miR-148a, miR-148b (that share an identical seed) and miR-152 [15]. A recent study by Zhu and colleagues showed that miR-152 down-regulates HLA-G expression by targeting its 3′UTR [19]. However, it is still unknown whether the C/G polymorphism at position +3142 of the gene can indeed affect HLA-G expression, whether the miRNA-mediated down-regulation of HLA-G has functional consequences (for example, effect on inhibitory receptor binding to HLA-G), whether miR-148 could also target HLA-G and what is the expression pattern of these miRNAs in the relevant tissue, the placenta.

Here we demonstrate that both miR-148a and miR-152 down-regulate HLA-G expression and that this down-regulation has functional consequences, as it prevents HLA-G-mediated inhibition of NK cell killing, through the LILRB1 inhibitory receptor. We further show that, contrary to what has been suggested, the C/G polymorphism has no influence on HLA-G regulation by these miRNAs. Finally, we show that the expression levels of the HLA-G-targeting miRNAs in the placenta are low as compared to other tissues and that the mRNA levels of HLA-G in the placenta are high. Therefore, we suggest that this might allow the expression of HLA-G in the placenta and not in other healthy tissues.

## Materials and Methods

### Cells

We used the RKO cell line (obtained from ATCC) and the MHC class I-negative human B lymphoblastoid cell line 721.221 [20]. Two transfectants were generated: 721.221 cells expressing HLA-G with a guanine nucleotide at position +3142 (with or without its 3′UTR). In addition, an additional transfectant was generated, expressing HLA-G with its 3′UTR with a cytosine nucleotide at position +3142 of the gene. To generate the 721.221/HLA-G transfectants, the HLA-G protein with its 3′UTR was amplified from JEG-3 cells (expressing guanine at position +3142, verified by sequencing compared to the published sequence, accession number NM_002127) using the following primers: Fw HLA-G primer: 5′ CGGGATCCGCCGCCACCATGGTGGTCATGGCGCCC 3′. Rv HLA-G +3′UTR primer: 5′GGCATTCAAAGTTCTCATGTCTTCCATTTA 3′.

The plasmid expressing HLA-G with a cytosine base in position +3142 in the 3′UTR was generated by PCR. First, the upstream fragment was amplified using the 5′ HLA-G edge primer, described above and an internal 3′ primer bearing the mutation (5′ tagctcagtggaccacaaatt 3′). The downstream fragment was amplified using an internal 5′ primer bearing the mutation (5′aatttgtggtccactgagcta 3′) and the 3′ HLA-G+3′UTR edge primer, described above. Next, both purified fragments were mixed together with the 5′ edge primer and the 3′ edge primer to generate the mutated full-length cDNA.

To generate a plasmid expressing HLA-G without its 3′UTR, the same Fw primer was used and the following Rv primer was used: 5′HLA-G GGAATTCTCAATCTGAGCTCTTCTTTCT 3′. The various fragments were then cloned into the pcDNA3 mammalian expression vector and stably transfected into the 721.221 cell line.

Peripheral blood lymphocytes were isolated from healthy female donors using Ficoll gradient. NK cells were further purified by using the human NK cell isolation kit and the autoMACS instrument (Miltenyi Biotec), according to the manufacturer's instructions. The identity of NK cells was confirmed by FACS. Purity was >98%. LILRB1-positive NK clones were grown in culture in the presence of 10 ng/ml IL-15 (PeproTech) and then used for NK cytotoxicity assays.

### Lentiviral constructs and transduction

The following oligonucleotides were used to generate artificial short hairpin RNAs that function as orthologs of pre-miRNA hairpins (the sequences of the mature miRNAs are in capital letters):

Fw miR148a: 5′gatccccTCAGTGCACTACAGAACTTTGTttcaagagaACAAAGTTCTGTAGTGCACTGAtttttggaaa 3′

Rv miR-148a: 5′agcttttccaaaaTCAGTGCACTACAGAACTTTGTtctcttgaaACAAAGTTCTGTAGTGCACTGA ggg 3′.

Fw miR-152:

5′gatccccTCAGTGCATGACAGAACTTGGttcaagagaCCAAGTTCTGTCATGCACTGAtttttggaaa 3′.

Rv miR-152: 5′agcttttccaaaaaTCAGTGCATGACAGAACTTGGtctcttgaaCCAAGTTCTGTCATGCACTGAggg 3′.

Fw miR-BART12: 5′ gatcccc TCCTGTGGTGTTTGGTGTGGTTttcaagaga AACCACACCAAACACCACAGGAtttttggaaa 3′

Rev miR-BART12: 5′ agcttttccaaaaa TCCTGTGGTGTTTGGTGTGGTT tctcttgaa AACCACACCAAACACCACAGGA ggg 3′

The specific oligonucleotides of the different miRNAs were annealed and inserted into the pTendoplasmic reticulum vector and then were excised from the vector together with the H1 RNA polymerase III promoter into the lentiviral vector SIN18-pRLL-hEFIp-EGFP-WRPE, as described [21]. In all experiments, miR-BART12 miRNA of EBV was used as the control microRNA. Lentiviruses were produced by transient three-plasmid transfection, as described [21]. These viruses were used to transduce the cell lines used, in the presence of polybrene (5 g/ml).

### Immunohistochemistry

The institutional review board of Hadassah–Hebrew University Medical Centers approved the use of decidual and placental waste material from healthy elective first-trimester pregnancy terminations, according to the principles of the Helsinki convention. Immunohistochemistry for HLA-G was performed on first trimester human placental formalin fixed paraffin embedded sections [22]. Briefly, we performed microwave antigen retrieval with sodium citrate buffer pH 6.0 (Zymed Laboratory Inc). Sections were incubated overnight at 4°C with either mouse anti-human 4H84 HLA-G antibody (kindly provided by Dr. Mike McMaster [23] or with a non-immune control serum. Detection was with anti-mouse Envision plus HRP (DakoCytomation) and AEC single solution (Zymed Laboratory Inc.). Sections were lightly stained with hematoxylin (BioGenex).

### Luciferase assay

For the firefly luciferase vector we used the pGL3 control vector (Promega). The 3′UTR of HLA-G (containing guanine or cytosine at position +3142) was amplified by PCR from the pcDNA3 plasmid expressing the HLA-G protein with its 3′UTR (described above) and inserted into XbaI site immediately downstream from the stop codon. The primers used for this amplification were:

Fw 3′UTR XbaI: 5′gctctagaattgaaaaggagggagctact 3′ and Rv 3′UTR XbaI: 5′gctctagaaagttctcatgtcttccattt 3′. For the generation of the mutated 3′UTR of HLA-G the seed binding region in the 3′UTR was mutated by PCR. First, the upstream fragment was amplified using the 5′ edge primer, used to amplify the 3′UTR and an internal 3′ primer bearing the mutation (mut middle: 5′agttatagcagtcgtgaccacaaa 3′). The downstream fragment was amplified using an internal 5′ primer bearing the mutation (mut middle 5′tttgtggtcacgactgctataact 3′) and the 3′ edge primer used to amplify the 3′UTR.

RKO cells were transduced with the lentiviral vectors for miR-148a, miR-152 and the control miRNA. Cells in 24-well plates were transfected with LT1 transfection reagent (MiRus) with 200 ng firefly luciferase reporter vector and 50 ng renilla luciferase pRL-CMV (control; Promega) in a final volume of 0.5 ml. Firefly and renilla luciferase activities were measured consecutively with the Dual-Luciferase Assay system (Promega) 48 h after transfection. This system allows us to estimate the down-regulation of the 3′UTR of HLA-G (which is cloned downstream to the firefly luciferase gene) by the miRNAs present in the transduced RKO cells. As an internal control, the firefly luciferase activity is normalized to renilla luciferase activity (both constructs are co-transfected to the cells and their activity is measured from a single sample). To finally determine the effect of the miRNAs on the 3′UTR of HLA-G, the normalized firefly luciferase activity, which was measured in the transduced cells expressing either miR-148a or miR-152, is further normalized to the firefly luciferase activity which was measured in the transduced cells expressing the control miRNA.

### FACS staining, antibodies and fusion proteins

The following antibodies were used: DyLight 649-conjugated goat anti-mouse (Jackson ImmunoResearch), mouse anti human HLA-G (clone MEM-G9, AbD Serotec), DyLight 649-conjugated goat anti-human IgG was used as a secondary Ab for the staining with the fusion protein. For staining of the NK clones, the mAb anti hILT2 (R&D systems) was used. The fusion protein used in the present study was LILRB1-Ig (CCM5-N-Ig, containing only the N-domain of CCM5, was used as a negative control [24]). The production and the purification of all fusion proteins were performed as previously described [25]. For FACS staining, the antibodies were used at concentrations recommended by the manufacturer. For staining with the fusion proteins, a concentration of 5 µg/well was used.

### Cytotoxicity assay

The cytotoxic activity of LILRB1-positive NK clones against target cells was assessed in 5-hour 35S-release assays, as described [25]. E∶T ratio was 1∶1.

### RNA and real-time quantitative PCR

For quantitative RT-PCR analysis of mRNA, cDNA was produced from a human total RNA survey panel from Ambion (AM6000). All specimens were polyadenylated with poly(A) polymerase (Ambion). RNA was reverse-transcribed with Moloney murine leukemia virus reverse transcriptase (Invitrogen) and 0.5 g poly(T) adaptor. DNA was amplified with specific primers and Platinum SYBR Green qPCR SuperMix-UDG with ROX (Invitrogen). Reaction primers were a 3′ adaptor primer and primers based on the microRNA sequences. The following primers were used in quantitative real-time PCR analysis of mRNA levels: Fw HLA-G: 5′ tgctgagatggaagcagtcttc 3′ Rv HLA-G: 5′ actacagctgcaaggacaacca 3′ FwUBC: 5′cagccgtatatcttcccagact3′ RvUBC: 5′ctcagagggatgccagtaatcta 3′ The following primers were used to quantify mature miRNA levels by quantitative realtime-PCR: miR-152: 5′ tcagtgcatgacagaacttgg 3′ miR-148a: 5′ tcagtgcactacagaactttgt 3′ miR-16: 5′ tagcagcacgtaaatattggcg 3′ hU6: 5′ atgacacgcaaattcgtgaag 3′.

## Results

### MiR-148a and miR-152 can potentially target the 3′UTR of HLA-G

**Figure 1:**
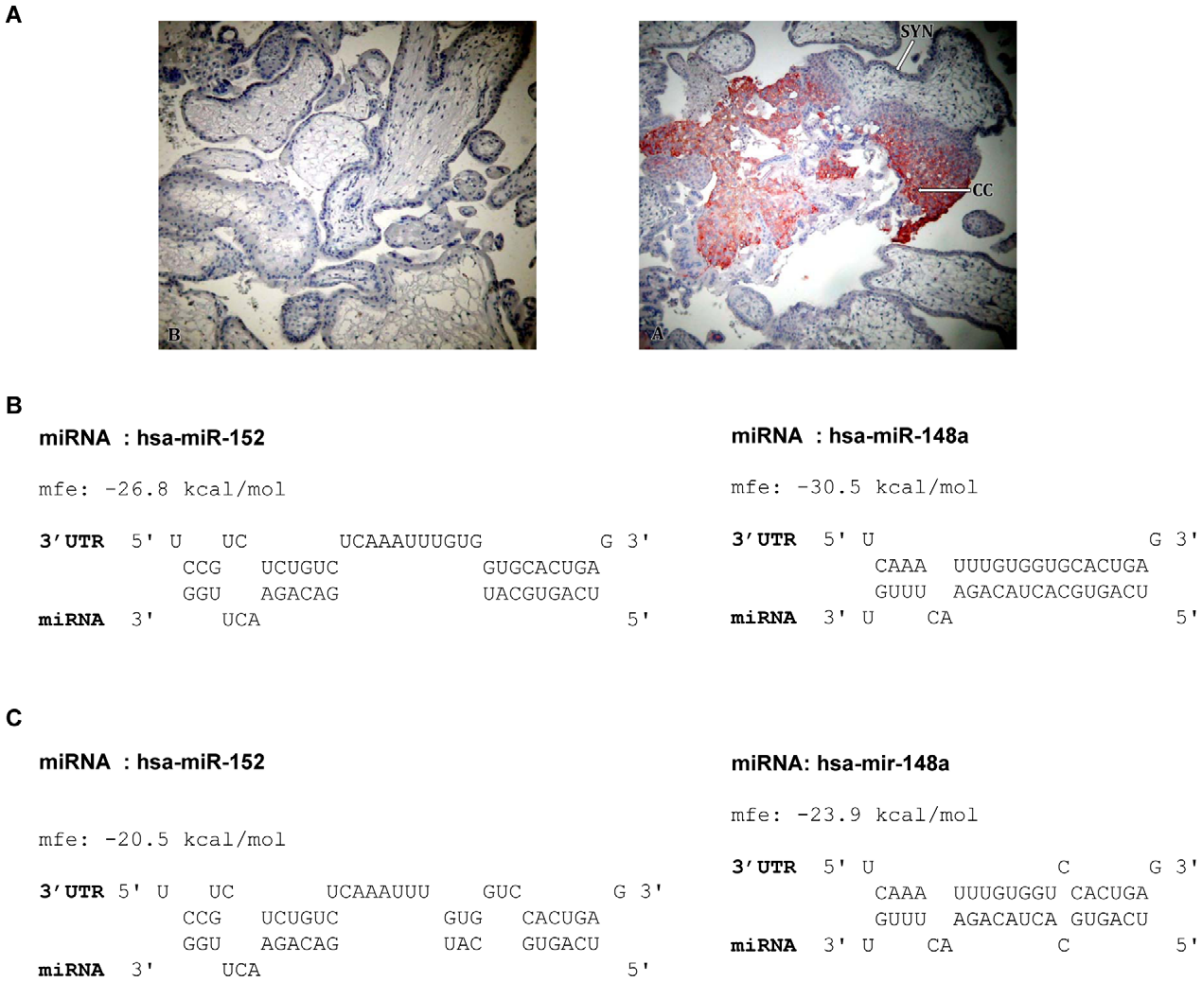
MiR-148a and miR-152 potentially target the 3′UTR of HLA-G. (A) Immunohistochemisty performed on first trimester placental tissue sections. Right panel: negative control. Left panel: HLA-G expression is detected in the trophoblast cell columns and extravillous trophoblasts. (B) Alignment of the 3′UTR of HLA-G (G variant) with the predicted miRNAs: miR-152 left panel, miR-148a right panel. (C) Alignment of the 3′UTR of HLA-G (C variant) with the predicted miRNAs: miR-152 left panel, miR-148a right panel.

Recently, several studies suggested that HLA-G expression is regulated by the cellular miRNAs miR-148a, miR-148b and miR-152 which have predicted binding sites in its 3′UTR [15], [19]. In addition, it was proposed that a C/G polymorphism at position +3142 affects the expression of HLA-G, such that the presence of a guanine nucleotide at this position was claimed to favor binding by these miRNAs [13], [15]. Because none of these studies examined the functional consequences of the down-regulation of HLA-G by the suggested miRNAs and since they did not examine whether the polymorphism at position +3142 indeed affects the miR-mediated regulation of HLA-G expression, we decided to investigate this. We have also studied the function of the HLA-G targeting miRNAs with regard to the interaction of HLA-G with NK cells, since under normal conditions the expression of HLA-G is restricted mainly to the EVT cells (figure 1A), where it plays an important role in immune cell modulation [26], [27].

To verify that a binding site for miR-148a, miR-148b and miR-152 exists in the 3′UTR of HLA-G we used the RNAhybrid algorithm [28]. Since the seed region of miR-148b is identical to that of miR-148a, we reasoned that the activity of these two miRNAs would probably be similar and we continued our predictions and later on the experimental analysis only with miR-148a.

On the basis of the calculation of minimum free energy (mfe) it can be indeed predicted that both miR-148a and miR-152 can potentially target the 3′UTR of HLA-G (figure 1B). The analysis further showed that as predicted, the binding of these miRNAs to the 3′UTR is less stable when cytosine is present at position +3142, because the seed region comprises only six base pair match, unusually starting from the first nucleotide (figure 1C).

### MiR-148a and miR-152 specifically target the 3′UTR of HLA-G

**Figure 2:**
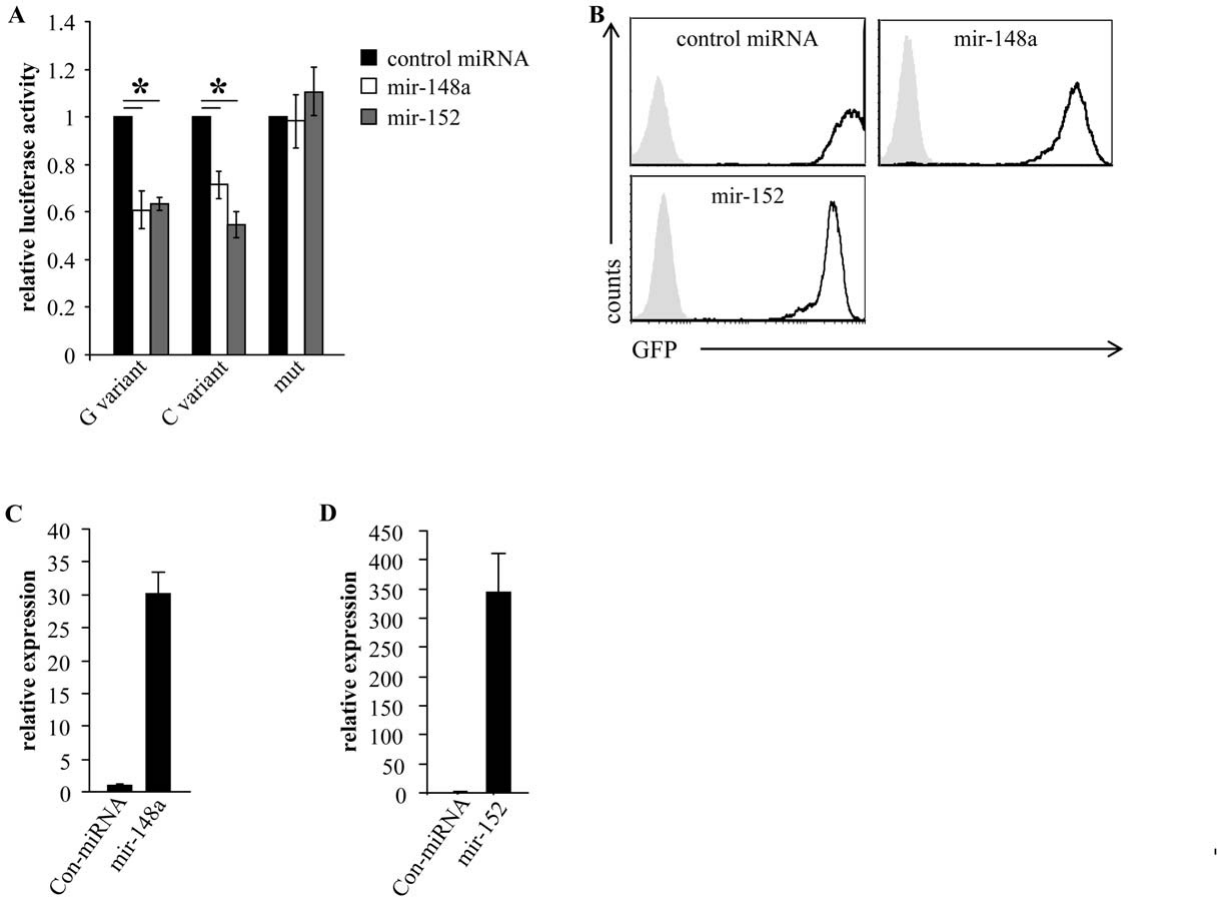
MiR-148a and miR-152 specifically target the 3′UTR of HLA-G. (A) Luciferase activity in RKO cells transduced with control miRNA (black columns), miR-148a (white columns) or miR-152 (grey columns). Results are presented relative to control reporter activity. G/C variant, reporter containing the WT sequence of the 3′UTR of HLA-G, which contains guanine or cytosine at position +3142, as indicated in the figure. Mut, reporter mutated at the seed sequence of HLA-G 3′UTR. Values are mean ± s.d. of triplicate samples. * *P*<0.05 (two-tailed Student's *t*-test). One out of three representative experiments is shown. (B) FACS histogram showing GFP levels (indicative for miRNA expression) of the miRNA-infected 721.221 cells. (C–D) Quantitative real-time PCR analysis of miR-148a (C) or miR-152 (D) in 721.221/HLA-G cells expressing the G variant transduced with the relevant miRNA or a control miRNA, as indicated in the figure. Results presented relative to U6. One out of three representative experiments is shown.

To demonstrate that miR-148a and miR-152 directly bind the 3′UTR of HLA-G, we performed a dual luciferase reporter assay. For this, we generated three firefly luciferase constructs: one contained the 3′ UTR of HLA-G with a guanine at position +3142 (G variant), one contained the 3′ UTR of HLA-G with a cytosine at position +3142 (C variant) and another in which the 3′UTR of HLA-G was mutated at the ‘seed sequence’ of the predicted miR-148a and miR-152 binding sites (the mutation included both binding sites). We transiently transfected these constructs into the RKO human colon cancer cells that had been transduced with miR-148a, miR-152 or a control miRNA. As can be seen in figure 2A, the luciferase activity was significantly repressed in cells expressing miR-148a or miR-152, irrespectively of whether G or C was present at the 3′UTR of HLA-G. The mutation at the seed sequence of the predicted site completely abolished the repression mediated by each miRNAs (figure 2A). These results indicate that the predicted binding sites were indeed targeted by both miR-148a and miR-152 and that the G/C polymorphism has no influence on miRNA-mediated targeting, despite the unusual seed match (figure 1C).

### MiR-148a and miR-152 down-regulate HLA-G expression

To demonstrate that miR-148a and miR-152 can target HLA-G we generated transfectants of 721.221 cells that express HLA-G with its 3′UTR (G or C variant, the expression of HLA-G on these transfectants is shown in the figures below). In addition, we cloned miR-148a, miR-152 and a control miRNA into lentiviral vectors that also contain a green fluorescent protein (GFP) cassette, which allowed us to monitor transduction efficiency. These constructs were then transduced into the 721.221/HLA-G transfectants. The lentiviral transduction was extremely efficient (figure 2B). We confirmed the expression of miR-148a and miR-152 in the transduced cells by quantitative real-time PCR (figure 2C and D). Although the analysis shows a much higher expression of miR-152 compared to mir-148a in the transduced cells, the base lines for each miRNA are not comparable, since different primer sets (having probably different binding efficiency) are used for the detection of each of the miRNA. Similar results were obtained with transfectants of the G and the C HLA-G variants (data not shown).

**Figure 3:**
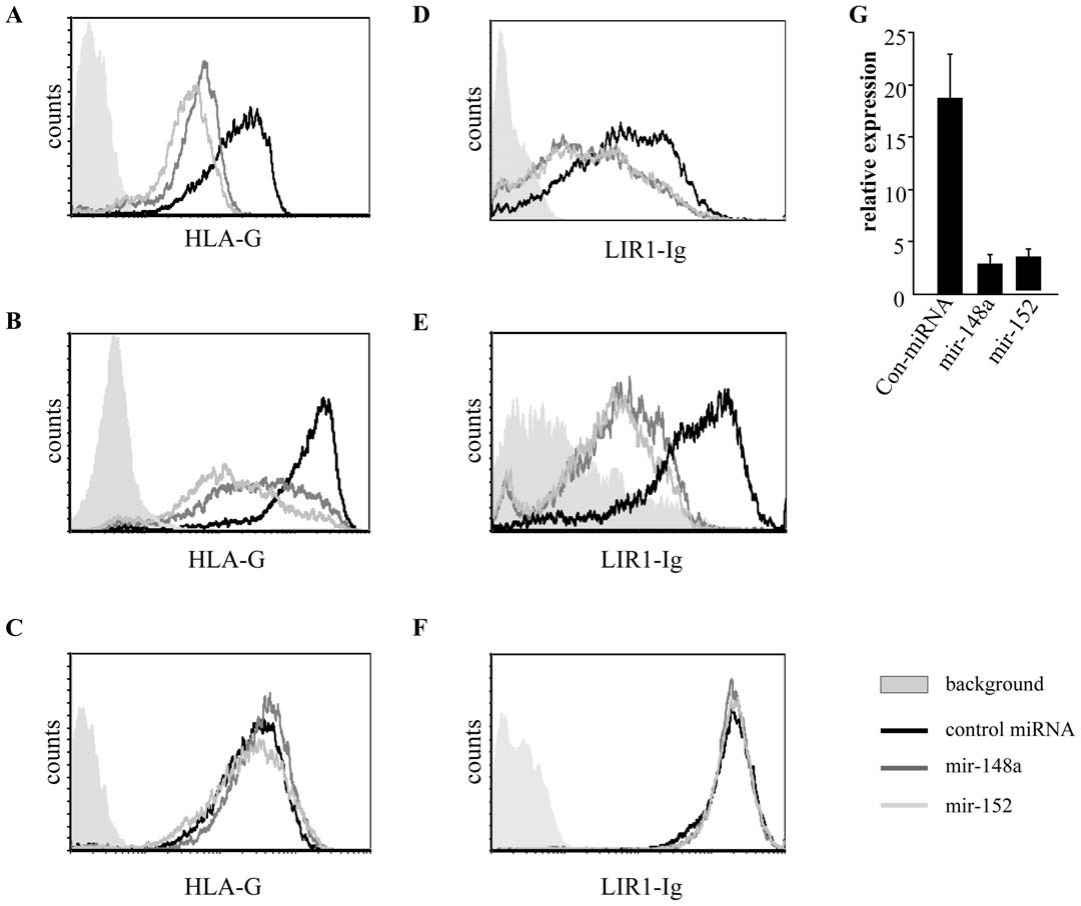
MiR-148a and miR-152 down-regulate HLA-G expression and reduce LILRB1 binding. 721.221/HLA-G (G variant, A, or C variant, B) were transduced with the control miRNA, miR-148a or miR-152. The various cells were then stained with anti HLA-G mAb and analyzed by FACS. One out of three representative experiments is shown. (C) FACS histograms re HLA-G staining of 721.221/HLA-G cells not expressing the 3′UTR of HLA-G. One out of three representative experiments is shown. (D–E) LILRB1-Ig staining of 721.221/HLA-G (G variant, D, or C variant, E) and of 21.221/HLA-G cells not expressing the 3′UTR of HLA-G (F) transduced with control miRNA, with miR-148a or with miR-152. Black histogram: cells transduced with a control miRNA. Dark grey histogram: cells transduced with miR-148a. Light grey histogram: cells transduced with miR-152. One out of three representative experiments is shown. (G) Quantitative real-time PCR analysis of HLA-G mRNA levels in 721.221/HLA-G cells (G variant), presented relative to hUBC. One out of three representative experiments is shown.

We next used our 721.221/HLA-G transfectants expressing HLA-G with or without its 3′ UTR and examined the expression levels of HLA-G in the presence or in the absence of miR-148a and miR-152 (figure 3). As can be seen in figure 3A and B, in the presence of both miRNAs, HLA-G expression (G and C variants) was down-regulated, compared to the control miRNA. Importantly, in the absence of the 3′UTR of HLA-G, no miRNA-mediated down-regulation of HLA-G expression was observed (figure 3C). Thus, both miR-148 and miR-152 directly target the 3′ UTR of HLA-G and such targeting lead to HLA-G down-regulation.

HLA-G is mainly recognized by the inhibitory receptor LILRB1 [6], [7] and although LILRB1 can bind also other MHC-I molecules, its affinity towards HLA-G is the highest [12], [29]. Therefore, to test whether the down-regulation of HLA-G by miR-148a and miR-152 would also lead to decreased binding of LILRB1 to the transduced cells, we performed FACS staining with LILRB1-Ig fusion protein (produced as described in [25]). As can be seen in figure 3D and E, in the presence of miR-148a or miR-152 the binding of LILRB1-Ig to 721.221/HLA-G cells, expressing the G variant, (figure 3D) or the C variant (figure 3E) was indeed decreased. In the absence of the 3′UTR of HLA-G the binding of LILRB1-Ig to the transduced cells was not changed (figure 3F).

We also transduced JEG-3 cells, which endogenously expresses HLA-G, with miR-148a and with miR-152 and, as expected, observed down-regulation of HLA-G (data not shown). However, we chose not to continue our experiments with this cell line, since it is not killed by NK cells as it does not express most of the killer ligands for the NK killer receptors [30].

To reveal the mechanism of the miRNA-mediated repression, we performed quantitative real-time PCR analysis of the 721.221/HLA-G cells transduced with either miR-148a, miR-152 or a control miRNA to evaluate the abundance of the HLA-G transcript. We observed that in the presence of both miR-148a and miR-152, the mRNA levels of HLA-G were substentially decreased (figure 3G). This suggests that the miRNAs' mode of action was mRNA degradation and not translational repression. The same results were obtained for both the G and the C variants (data not shown).

### Functional implications

**Figure 4:**
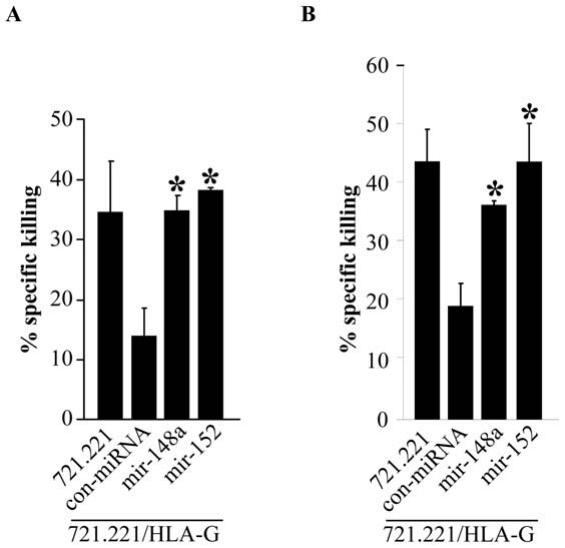
The miRNA-mediate reduction of HLA-G expression enhances NK cell cytotoxicity. (A–B) 721.221 and 721.221/HLA-G cells, expressing either the G variant (A) or the C variant (B), transduced with control miRNA, miR-148a or miR-152, were used as target cells in killing assays against NK clones expressing LILRB1. The effector: target (E∶T) ratio was 1∶1. Values are mean ± s.d. for triplicate samples. * *P*<0.02 (two-tailed Student's *t*-test). One out of three representative experiments is shown.

The interaction between LILRB1 and HLA-G leads to inhibition of NK cell-mediated cytotoxicity [12]. Therefore, to test whether the miRNA-mediated down-regulation of HLA-G expression would affect NK cell cytotoxicity, we isolated LILRB1-expressing NK clones and tested their activity in cytotoxicity assays directed against the various 721.221/HLA-G transduced cells. As can be seen in figure 4, the parental class I MHC negative 721.221 cells [20] were killed by the NK clones. In the presence of a control miRNA in the 721.221/HLA-G cells, HLA-G was not down-regulated (figure 3) and indeed a significant inhibition of NK cytotoxicity could be seen. Importantly, in the presence of miR-148a or miR-152, when HLA-G was down-regulated (figure 3), the HLA-G inhibition was significantly abolished, irrespectively of whether the transfected cells expressed the C (figure 4A) or the G (figure 4B) variant. Thus, the miR-148a and 152 targeting of HLA-G resulted in reduced HLA-G expression, reduced LILRB1-Ig binding and consequently, reduced HLA-G-mediated inhibition of NK killing.

### Expression of miR-148a, miR-152 and HLA-G in the placenta

**Figure 5:**
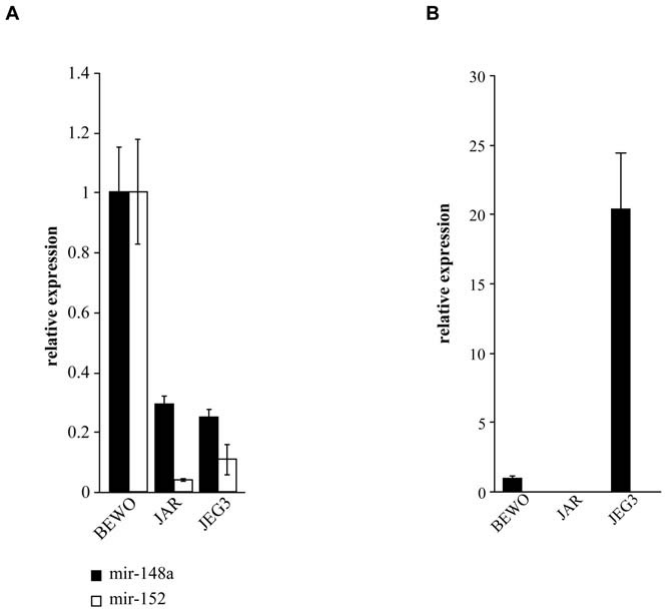
Expression of miR-148a, miR-152 and HLA-G mRNA in choriocarcinoma cell lines. (A) Quantitative real-time PCR analysis for miR-148a (black columns) and miR-152 (white columns) in BEWO, JAR and JEG-3 cell lines, presented relative to U6. (B) Quantitative real-time PCR analysis of HLA-G mRNA in BEWO, JAR and JEG-3 cell lines, presented relative to hUBC. One out of three representative experiments is shown.

EVT cells in the placenta express high levels of HLA-G ([4] and figure 1A). Our final aim was to test whether the expression of the HLA-G targeting miRNAs and the HLA-G mRNA would be different among various tissues and cell lines. For that purpose, we initially determined whether the HLA-G targeting miRNAs are expressed in three choriocarcinoma cell lines; JEG-3 (HLA-G positive), BEWO and JAR (HLA-G negative, data not shown) and observed that the expression of miR-148a and miR-152 were higher in the BEWO cell line, compared with the JAR and JEG-3 cell lines (figure 5A). In both the JAR and BEWO cell lines, however, the mRNA of HLA-G could hardly be detected (figure 5B). In contrast, in the HLA-G-expressing JEG-3 cells HLA-G mRNA was present (figure 5B). Thus, we concluded that HLA-G is expressed in JEG-3 cells and not in BEWO and JAR cells not because of the miRNA activity but rather because the later two cell lines express little or no HLA-G mRNA.

**Figure 6:**
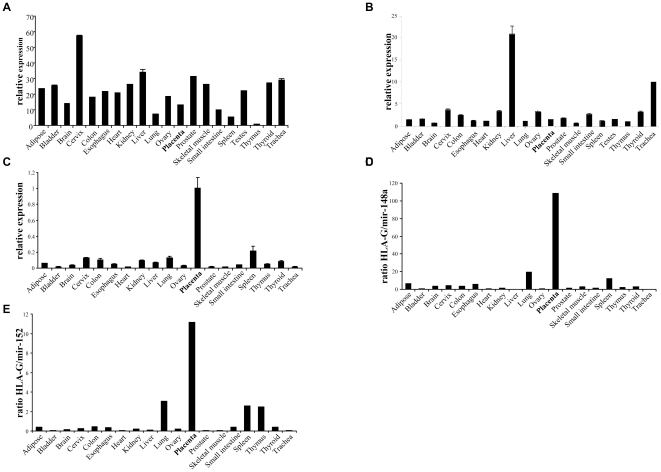
Expression of miR-148a, miR-152 and HLA-G mRNA in various human tissues. (A–C) Quantitative real-time PCR analysis of miR-148a (A), miR-152 (B) and the mRNA of HLA-G (C) in different healthy tissues, presented relative to miR-16 (A and B) or hUBC (C). (D–E) Ratio of HLA-G mRNA levels divided by the miR-148a levels (D) or by miR-152 levels (E). One out of three representative experiments is shown.

We next evaluated the expression of the HLA-G mRNA and miRNAs in various human tissues by using quantitative real-time PCR. Because the mRNA levels are probably different among various tissues, an internal normalizer control must be used. For that purpose mir-16 was used as a reference miRNA, since the expression of this miRNA is quite ubiquitous in almost all somatic tissues. As can be seen in figure 6A and B, miR-148a and miR-152 were expressed at relatively low levels in the placenta, compared to other healthy tissues and as expected, the mRNA levels of HLA-G were particularly high in the placenta (figure 6C). Because efficient targeting of a particular gene by miRNAs would happen if the relevant miRNA is found in sufficient access, we calculated the expression ratios of HLA-G mRNA and its targeting miRNAs. As can be seen in figure 6D and E (for miR-148a and miR-152, respectively), the ratio of the expression of HLA-G mRNA relative to its targeting miRNAs was the highest in the placenta. This can be one of the reasons explaining why HLA-G expression is detected under normal conditions almost exclusively in the placenta.

## Discussion

HLA-G is a non-classical MHC-Ib molecule with a restricted distribution that is almost completely confined to the EVT cells of the fetus [4]. As an immunomodulatory molecule [12], [29], HLA-G possesses several unique features, one of which is the inhibition of decidual NK cell cytotoxicity against the fetal cells [4]. These decidual NK cells are the dominant lymphocyte population at the fetal-maternal interface during the first trimester [10], [31], [32].

It has been suggested that the expression of HLA-G is controlled by miRNAs, which target the 3′UTR of HLA-G mRNA, thereby down-regulating its expression [15]. Three miRNAs were suggested to be involved in the HLA-G targeting: miR-148a, miR-148b and miR-152 [15], [19]. In addition, it was proposed that a C/G polymorphism at position +3142 in the 3′ UTR of HLA-G might affect the efficiency of the miRNA targeting of HLA-G [13], [15]. However, the study by Tan and colleagues [15] focused on soluble HLA-G and not membrane bound- HLA-G and the recent study by Zhu XM and colleagues [19] investigated the function of miR-152 only. Thus, many questions still remain unanswered. First, there is no information regarding miR-148. Second, the mechanism of action of these miRNAs is not determined. Third, proper functional cytotoxicity assays involving primary human NK cells were not performed [6], [7]. In addition, these studies did not test whether the HLA-G targeting miRNAs are expressed in the placenta and whether the 3′ UTR polymorphism is really important for the miRNA regulation. In this manuscript we provide answers to these questions.

We show here that both miR-148a and miR-152 target HLA-G at its 3′UTR and hence down-regulate HLA-G expression. This down-regulation of HLA-G expression has functional consequences, as it diminished the binding of the inhibitory receptor LILRB1 to the transduced cells and consequently abolished the HLA-G mediated inhibition of NK cytotoxicity. Furthermore, we have found that the C/G polymorphism has no influence on HLA-G expression. Interestingly, while we show that the +3142 C/G polymorphism makes no difference to the miR inhibition of HLA-G, a different polymorphism at the 3′UTR of HLA-C did show an effect on miR-148a inhibition of HLA-C [33].

We further show the physiological implications of the miR-148a and miR-152 mediated down-regulation of HLA-G expression. As mentioned above, HLA-G expression is almost completely restricted to the EVT cells [4]. Therefore, we determined the miRNA levels and the mRNA levels of HLA-G in three choriocarcinoma cell lines: JEG-3 (expressing HLA-G), JAR and BEWO (lacking HLA-G expression) and observed that the expression of HLA-G in these cell lines is probably dependent on its mRNA levels. Analysis of the miRNA levels in the placenta showed very low levels of both miR-148a and miR-152, compared with other healthy tissues. Moreover, the ratio of HLA-G mRNA expression relative to its targeting miRNAs is the highest in the placenta and this might explain the almost exclusive expression of HLA-G in the placenta.

Several findings suggest that the regulation of HLA-G by miR-148a and miR-152 extends beyond normal physiological conditions. It has been suggested that HLA-G is important for a successful pregnancy not only due to its protective effect on the fetal cells (by inhibiting immune cell function) but also due to its effect on cytokine and angiogenic factor secretion by NK cells [9], [10]. Low HLA-G expression levels at the fetal-maternal interface were associated with pregnancy complications, such as preeclampsia [11] and interestingly, higher levels of miR-152 were detected in preeclamptic placentas and high levels of miR-148a were detected in preterm labor placenta [16], [34]. In addition, HLA-G expression is up-regulated in tumors [13] and low levels of miR-152 and miR-148a were found in tumors, such as breast cancer and gastrointestinal cancers [13], [35]. Nevertheless, although miR-148a and mi-152 are clearly important in regulating HLA-G expression in normal tissue and although studies suggested strong association of both miRNAs and HLA-G with the mentioned pathologies, further studies are needed to determine the actual involvement of these miRNAs in pathological conditions.

In summary, our study shows that both miR-148a and miR-152 specifically regulate HLA-G expression by targeting its 3′UTR and that this regulation may control NK-mediated killing, through the down-regulation of HLA-G.
